# Magnesium in Kidney Function and Disease—Implications for Aging and Sex—A Narrative Review

**DOI:** 10.3390/nu15071710

**Published:** 2023-03-31

**Authors:** María del Carmen Macías Ruiz, Lorena Cuenca Bermejo, Nicola Veronese, Emiliano Fernández Villalba, Ana María González Cuello, Karolina Kublickiene, Valeria Raparelli, Colleen M. Norris, Alexandra Kautzky-Willer, Louise Pilote, Mario Barbagallo, Ligia Dominguez, María Trinidad Herrero

**Affiliations:** 1 Clinical and Experimental Neuroscience (NiCE), Institute for Aging Research, Biomedical Institute of Murcia (IMIB-Pascual Parrilla), School of Medicine, Campus Mare Nostrum, UniWell, University of Murcia, 30100 Murcia, Spain; 2 Geriatric Unit, Department of Medicine, University of Palermo, 90100 Palermo, Italy; 3 Department of Renal Medicine, Institution for Clinical Science, Intervention and Technology, Karolinska Institute, 17177 Stockholm, Sweden; 4 Department of Translational Medicine, University of Ferrara, 44121 Ferrara, Italy; 5 Faculty of Nursing, University of Alberta, Edmonton, AB T6G 2R3, Canada; 6 Cardiovascular and Stroke Strategic Clinical Network, Alberta Health Services, Edmonton, AB T5J 3E4, Canada; 7 Division of Endocrinology and Metabolism, Department of Medicine III, Medical University of Vienna, 1090 Vienna, Austria; 8 Research Institute of McGill University Health Centre, Divisions of General Internal Medicine and Clinical Epidemiology, McGill University, Montreal, QC H4A 3J1, Canada; 9 Faculty of Medicine and Surgery, University of Enna “Kore”, 94100 Enna, Italy

**Keywords:** magnesium, elderly, kidney function, chronic kidney disease, sex

## Abstract

Magnesium (Mg) has a vital role in the human body, and the kidney is a key organ in the metabolism and excretion of this cation. The objective of this work is to compile the available evidence regarding the role that Mg plays in health and disease, with a special focus on the elderly population with chronic kidney disease (CKD) and the eventual sex differences. A narrative review was carried out by executing an exhaustive search in the PubMed, Scopus, and Cochrane databases. Ten studies were found in which the role of Mg and sex was evaluated in elderly patients with CKD in the last 10 years (2012–2022). The progression of CKD leads to alterations in mineral metabolism, which worsen as the disease progresses. Mg can be used as a coadjuvant in the treatment of CKD patients to improve glomerular filtration, but its use in clinical applications needs to be further characterized. In conclusion, there’s a need for well-designed prospective clinical trials to advise and standardize Mg supplementation in daily clinical practice, taking age and sex into consideration.

## 1. Introduction

Magnesium (Mg) is one of the most important cations in the human body, but it has not been fully considered in preclinical and clinical fields. Indeed, the pathophysiological and clinical importance of Mg has only recently been acknowledged. Mg is an essential element in energy storage and utilization. This essential cation is also involved in the synthesis, degradation, and polymerization of deoxyribonucleic acid, ribosome binding to ribonucleic acid, and protein synthesis [1].

The circulating concentrations of magnesium are regulated under normal physiological conditions and are maintained in a narrow range (0.75–0.95 mmol/L). This is managed mainly by its intestinal absorption and renal excretion [1,2].

Mg has special clinical relevance in states of decompensated cirrhosis, renal failure, intestinal malabsorption syndrome, prolonged high-dose diuretic therapy, acute pancreatitis, and extensive burns [3], among others.

### 1.1. Magnesium Metabolism

Mg is the second most frequent cation within the cell [4]. Further, it can be categorized into three fractions: ionized (55–70%), protein-bound (20–30%), and complexed with anions such as phosphate, bicarbonate, citrate, or sulfate (5–15%). Both the ionized and complexed Mg are the ultrafilterable fraction of Mg, which represents the portion of total serum Mg that can be excreted by the kidney [5].

**Figure 1:**
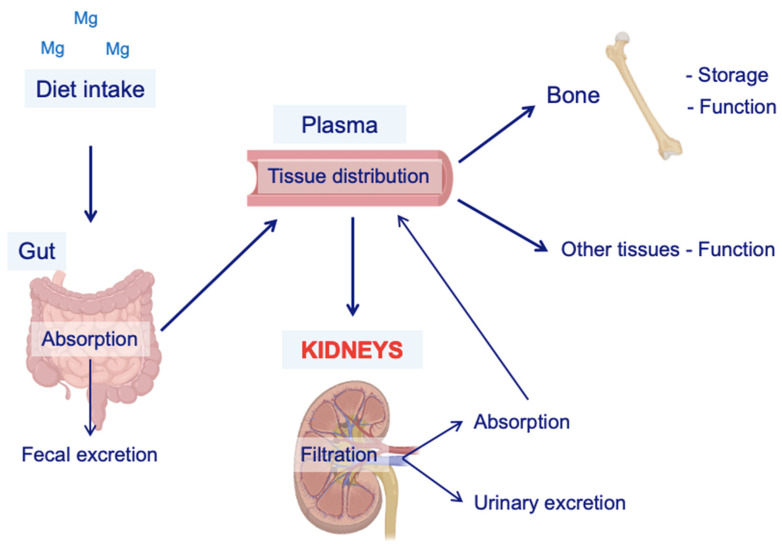
Schematic representation of Mg metabolism. The main source of Mg is diet intake. Once in the body, a fraction of the Mg contained in the food is absorbed by the gut, and the rest is eliminated via fecal excretion. Then, Mg is incorporated into the plasma to be distributed to the other organs for self-consumption and storage (e.g., the bones). Kidneys are the key regulators of Mg balance because of their ability to retake or excrete it, depending on the demands of the body. Mg = magnesium.

Mg can be stored in different forms (Figure 1). The bone storage of Mg is intimately associated with carboxyl apatite crystals, while the intracellular Mg reservoir is bound to lipoproteins, nucleoproteins, ribonucleic acids, and adenosine diphosphate [5,6]. 20% of the total Mg is in the plasma, bound to the plasma proteins. Thus, Mg concentrations are minimally affected by the variations in proteinemia, contrary to what happens with calcium [7]. Furthermore, the low amount of Mg in the extracellular fluid vs. intracellular and bone deposits explains why serum Mg is not a parameter of absolute reliability for assessing the state of Mg depletion or repletion [8].

Mg balance is highly dependent on dietary absorption. The adult daily Mg requirement is estimated to be 200–400 mg, and the dietary recommendation is 320 mg/day for women and 420 mg/day for men [9]. This cation is present in almost all foods and is especially abundant in green leafy vegetables because it is part of the composition of chlorophyll [10,11]. Intestinal Mg absorption mainly occurs in the distal small intestine, specifically from the distal duodenum (D3) to the ileum, by two reverse flows: a facilitated flow of transcellular absorption and another of passive paracellular secretion (Figure 1). On the other hand, the colon has a limited capacity to absorb Mg [11,12].

In healthy adults, Mg balance depends primarily on adjusting the renal Mg excretion to the intestinal absorption of the cation. This balance should be adjusted to maintain or restore the body’s Mg content within the physiological ranges [13].

**Figure 2:**
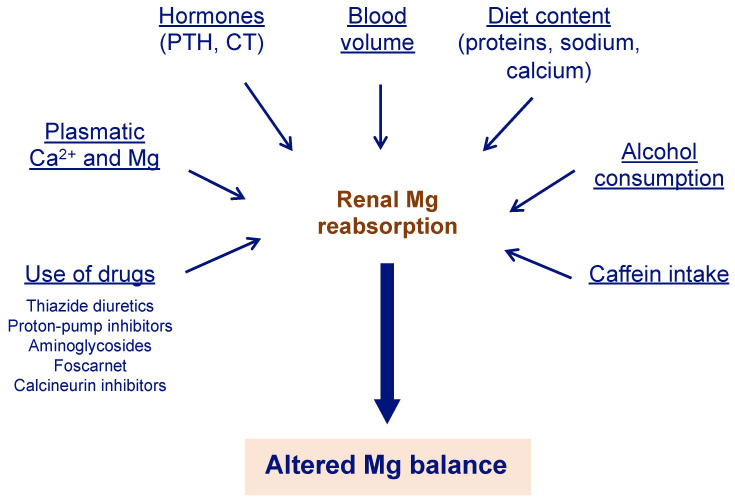
Different factors can alter renal Mg reabsorption. Mg absorption in the kidneys is a crucial step to ensure Mg balance. Therefore, different causes that alter this step can cause impaired Mg metabolism, with hypomagnesemia being the most common alteration. Mg can be obtained from legumes, dark and green leafy vegetables, nuts, seeds, whole grains, and fortified cereals, among others [9]. Ca^2+^ = calcium; CT = calcitonin; Mg = magnesium; PTH = parathyroid hormone.

Several factors can alter Mg balance, leading to hypermagnesemia or hypomagnesemia (Figure 2) [14]. Hypomagnesemia is much more common and does not present specific symptoms until the deficiency is severe [15,16].

Moderate hypomagnesemia is defined in a range of 0.50–0.65 mmol/L Mg and is usually asymptomatic, whereas severe and symptomatic hypomagnesemia appears when serum Mg levels are below 0.5 mmol/L [17]. The magnesiuria measurement helps in determining whether the origin of hypomagnesemia is renal (magnesiuria greater than 2 mmol/24 h) or extra-renal (magnesiuria less than or equal to 1 mmol/24 h) [16].

### 1.2. Kidney Function in Relation to Magnesium and Other Implicated Electrolytes

The kidney has a vital function in the regulation of Mg balance. Mg is filtered in the glomerulus and reabsorbed in the proximal tubule via a paracellular pathway in the loop of Henle [18]. The loop of Henle is a segment with reduced permeability to water, so its reabsorption is carried out through the paracellular route, which has a selective permeability to calcium and Mg. Furthermore, this process is facilitated by the binding proteins, claudin 16 and claudin 19 [19].

The distal convoluted tubule is also an essential segment for the regulation of renal Mg reabsorption. In this segment, 10% of the filtered Mg load is reabsorbed, which allows for adjustment of the final excretion of this cation according to the needs of the organism [20]. In this location, the reabsorption of Mg is independent of sodium reabsorption (transcellular) and involves molecular pathways of transport different from those of calcium [19].

The main determinants of renal reabsorption of Mg include variations in the normal levels of calcium and blood volume, which modify the reabsorption in the kidney of water and solutes, and hence of Mg. In other words, hypervolemia inhibits its reabsorption, while hypovolemia stimulates it [19,21]. In addition, hypermagnesemia and hypercalcemia inhibit the reabsorption of Mg in the loop of Henle by stimulating the calcium-sensing receptor (CaSR) [21].

Parathyroid hormone (PTH) and other hormones, capable of activating the cAMP pathway, stimulate the reabsorption of calcium in the kidney. There are no known hormones that particularly regulate Mg but PTH and calcitonin (CT) are known to have a positive effect on Mg balance (Figure 1) [22]. This effect may be related to the hypermagnesemic tendency of patients with hyperparathyroidism [23].

### 1.3. Kidney Disease and Magnesium

As mentioned, the kidney has a vital role in maintaining a normal concentration of Mg. Furthermore, when the glomerular filtration rate falls, the kidney’s ability to excrete Mg decreases accordingly [24].

In chronic kidney disease (CKD), there is a tendency towards hypermagnesemia, but it depends on the severity of the disease. For example, in CKD stages 1–3, an increase in fractional Mg excretion compensates for the loss of renal function, and, as a consequence, Mg levels remain within normal ranges. However, in advanced CKD (stages 4–5), compensatory systems are not sufficient, and the fraction of filtered Mg excreted increases as a result of impaired tubular reabsorption [25]. This becomes more evident when the glomerular filtration rate drops below 10 mL/min. In other words, the compensatory increase in fractional excretion of Mg is inadequate to prevent and increase serum Mg concentrations [26].

CKD patients in treatment with dialysis have both ionized and total Mg concentrations that tend to be higher than normal but always depend on the degree of residual kidney function [13]. However, patients with end-stage renal disease who are on treatment with dialysis usually have normal levels or can sometimes present hypomagnesemia. This could be a consequence of the diet, drug side effects (Figure 2) [14], or dialysate Mg concentration [27].

Additionally, it is common for CKD patients to have impaired intestinal Mg absorption compared to healthy individuals [28].

It has been previously shown that CKD patients with hypomagnesemia have a higher risk of increased vascular calcification and consequently increased cardiovascular risk, leading to a higher risk of mortality in dialysis patients [29,30,31]. On the other hand, hypomagnesemia is commonly related to patients that endure renal transplantation, as a consequence of the immunosuppressors that are used in the treatment to avoid graft failure [32]. Other authors have found a relationship with new-onset diabetes after renal transplantation, making a connection with patients that have post-transplant hypomagnesemia [15,33,34,35].

### 1.4. The Aging Kidney

In world demographics, life expectancy continues to increase; therefore, it is essential to consider the aging process as a variable in clinical and preclinical research. In particular, aging has a pivotal role in the structural and functional changes that occur in the kidney during our lives [36]. For example, the kidney experiences a progressive functional decline along with aging, as well as macroscopic and microscopic histological alterations, which can be accentuated by systemic comorbidities or by pre-existing kidney disease [37].

At the macroscopic scale, there is the formation of simple renal cysts and roughness of the kidney surface [38]. Even if cysts are formed in one or both kidneys, they usually do not cause enlargement of the organ [39,40]. These renal cysts have been correlated with hypertension, decreased renal size, and functional changes [41,42].

Kidney volume is an important indicator of renal dysfunction, and it is demonstrated to progressively decline with age. For example, the study conducted by Roseman and collaborators confirmed that kidney volume decreased by approximately 16 cm^3^ per decade over the age of 60 years [43]. Likewise, Wang and colleagues reported that kidney volume reduces by 22 cm^3^ per decade over the age of 50 years, in addition to a reduction in parallel of the renal cortex [44].

Microscopic changes associated with aging include nephrosclerosis, thickening of the glomerular basement membrane, mesangial widening, and increased extracellular matrix accumulation in aging kidneys [45].

Some reports showed that by the age of 30, the kidney losses approximately 6000–6500 nephrons each year, which has been correlated with the annual reduction of the glomerular filtration associated with the aging process [11,37]. Although it has been less studied, there is evidence suggesting that renal tubular function also declines progressively with aging [46].

Kidney function is a vital predictor of longevity, and the age-related decline in renal function shows several consequences for the quality of life [47]. Due to a gradual deterioration of the renal function reserve, kidney aging increases the risk of acute kidney injury and CKD [48,49]. Furthermore, 65-year-old patients (or older) are at higher risk of end-stage renal disease and drug-related nephrotoxicity [50].

### 1.5. Magnesium Alterations in Older Population

A relationship between decreasing Mg serum levels and aging has been reported. However, it is not clear whether this association is with the aging process or with the presence of diseases or pathological alterations in kidney function [50].

Several studies have shown that, worldwide, the average dietary intake is often inadequate or significantly lower, while the Mg requirements for body functioning do not change with age [51,52,53].

Aging seems to be a risk factor for inadequate Mg levels due to reduced intestinal absorption, and this could be related to the decrease in vitamin D levels [54]. Another reason that supports this statement is the increased urinary excretion of Mg [45]. As mentioned earlier, with advanced age, renal function and tubular reabsorption decline [46]. In addition, there are other factors that influence the decrease in Mg in elderly patients related to comorbidities and polypharmacotherapy [55]. For example, diuretic therapy may cause excessive urinary loss of Mg, and diuretic-induced hypomagnesemia is often accompanied by hypokalemia [14].

Hypomagnesemia may be present in about 40% of patients with hypokalemia, and correction of the Mg deficit is required to achieve correction of the potassium deficit [56]. As a result, it is recommended to assess Mg levels in patients with hypokalemia [11]. Some therapies that are commonly used in older adults can also contribute to Mg deficit (Figure 2) [14]. For example, proton-pump inhibitors are widely used by patients and prescribed by physicians and are highly toxic to the kidneys, causing hypomagnesemia, which, in turn, can lead to acute kidney injury that can progress to chronic kidney disease and worsen if the disease is already established [57].

## 2. Materials and Methods

The first searches were carried out in June 2022, combining the terms ‘magnesium’ and ‘ageing’ in the PubMed and Scopus databases.

Later, it was expanded with a combination, using the Boolean operators “AND” and “OR” as appropriate, of the terms ‘kidney function’, ‘hypermagnesemia’, ‘hypomagnesemia’, ‘gender’, ‘sex’, ‘ageing’, ‘elderly’, ‘geriatric patient’, and ‘elderly’. These searches yielded a considerable number of results, quite a few of which repeated or were not very useful for review, but they gave us an overview of the breadth of the theme.

During the search process, different databases were consulted (PubMed, Scopus, and Cochrane), of which the articles corresponding to the PICO search and through the MeSH (Medical Subject Headings) were taken into account.

The combination of terms that yielded the best results in all search engines was as follows: (Magnesium) AND (kidney disease): (“magnesium”[MeSH Terms] OR “magnesium”[All Fields] OR “magnesium s”[All Fields] OR “magnesiums”[All Fields]) AND (“kidney diseases”[MeSH Terms] OR (“kidney”[All Fields] AND “diseases”[All Fields]) OR “kidney diseases”[All Fields] OR (“kidney”[All Fields] AND “disease”[All Fields]) OR “kidney disease”[All Fields]). Magnesium: “magnesium”[MeSH Terms] OR “magnesium”[All Fields] OR “magnesium’s”[All Fields] OR “magnesiums”[All Fields] kidney disease: “kidney diseases”[MeSH Terms] OR (“kidney”[All Fields] AND “diseases”[All Fields]) OR “kidney diseases”[All Fields] OR (“kidney”[All Fields] AND “disease”[All Fields]) OR “kidney disease”[All Fields].

PICO question: P: Elderly with chronic kidney disease, I: role of Mg, C: sex O: improvement in kidney function.

The aim of this study was to point out the importance of a better understanding of the role of Mg in the elderly population with CKD and the possible differences by sex.

The inclusion criteria were: (a) observational studies or clinical trials that evaluated older patients with kidney disease, taking the role of magnesium into consideration, and (b) studies published in the period 2012–2022.

The exclusion criteria were: (a) articles outside the study period; (b) articles that included variables outside the scope of this study; (c) articles that did not include elderly patients; (d) systematic reviews or articles under review; and (e) articles in a language other than Spanish or English.

The search process involved the consultation of 1482 articles, of which 10 were finally selected.

## 3. Results

After the exhaustive search, 10 articles were found according to the conditions applied. As shown in Table 1, the 10 articles selected for this review found a positive relationship between high Mg levels in serum and a decreased risk of CKD-related mortality or cardiological complications. Furthermore, Sakaguchi and collaborators found an association between lower Mg levels and (i) increasing age, (ii) lower levels of albumin, calcium, phosphate, and hemoglobin, (iii) higher levels of C-reactive protein and alkaline phosphate, and (iv) a higher prevalence of diabetes mellitus, a history of cardiovascular disease, and hip fracture [58].

Moreover, telomere attrition has been demonstrated to be more prevalent in patients enduring dialysis and has a strong relationship with chronic systemic inflammation [59,60], which is considered the cause and consequence of the senescence in CKD [61], as well as with oxidative stress [62].

In addition, the protective effect of Mg intake on renal function in CKD patients is well established in all articles except one [63]. However, since the effect was not statistically significant, the low Mg group had a slightly more negative or detrimental monthly eGFR slope than the control group.

The relationship between treatment and Mg concentration was just shown in one study [64], which found an association with decreased Mg for proton-pump inhibitors and calcium supplementation. In particular, a higher concentration of Mg was found in the case of calcitriol. Furthermore, loop diuretics had a positive association (statistically significant) with serum Mg, whereas thiazide diuretics had an inverse association and potassium-sparing diuretics had no significant association.

**Table 1:** Characteristics of the studies included in this review are divided into sections relevant to the purpose of this study. Abbreviations: Magnesium (Mg), chronic kidney disease (CKD), Estimated Glomerular Filtration Rate (eGFR), Modification of Diet in Renal Disease Equation (MDRD), Chronic Kidney Disease Epidemiology Collaboration equation (CKD-EPI).

Authors	Year	Type of Study	Mean Age or Range and Sex	Population	Date	Measures	Results
Kanbay et. al. [65]	2012	Observational cohort study.	- 51 years. - Stage 3: (29–71 years, Male/Female: 48/53). - Stage 4: (31–73 years, Male/Female: 46/40). - Stage 5: (28–71 years, Male/Female: 45/51).	- 283 patients (101 in stage 3, 86 in stage 4, and 96 in stage 5). Of the Renal Unit of the Gulhane School of Medicine Medical Center, Ankara, Turkey	- Between March 2006 and December 2010. - The mean follow-up period was 38 months.	- eGFR was calculated using the MDRD (Modification of Diet in Renal Disease). - eGFR was treated as a continuous variable.	- Strong positive correlation between flow-mediated dilation and Mg values. - A higher Mg level is associated with less endothelial dysfunction (*p* < 0.001). - A higher level of Mg may protect against endothelial damage and is associated with better survival.
Wyskida et. al. [66]	2012	Prospective, open-label, cross-sectional clinical study.	- 54.4 ± 14.6 years. - 58 males and 43 females. - Control group: 10 males and 10 females.	- 101 hemodialysis patients and 20 patients with normal kidney function. - Katowice, Poland.	- Between 2011 and 2012.	- Hemodialysis three times per week for 4 to 5 h (12.7 ± 1.1 h weekly). - **Carbohydrate dialysate fluid** containing: **0.75 mmol/L of Mg** and polysulfone or cuprofane dialysis membranes was used. - Control group: mean serum Mg concentration was 0.89 ± 0.06 mmol/L.	- The average serum Mg concentration before hemodialysis was 1.32 ± 0.18 mmol/L, which was 48% higher than in the control group. - Hypermagnesemia (≥1.5 mmol/L) was found in 81.2% of hemodialysis patients. - With a higher prevalence in males (odds ratio = 1.98 [0.64 to 6.13], *p* = 23). - Strong positive correlation between daily intake of Mg and its serum concentration in hemodialysis patients (r = 0.870, *p* < 0.001).
Van Laecke et. al. [67]	2013	Retrospective cohort study.	- 57.4 ± 17.3 years - 56.1% were male.	- 1650 patients (stage 1: 22%; stage 2: 31.7%; stage 3a: 18.1%; stage 3b: 16.2%; stage 4: 10.8%; and stage 5: 1.2%). - Outpatient clinic of the nephrology unit of the tertiary university hospital.	- Between January 2002 and June 2011. - A median follow-up of 5.1 years.	- eGFR was calculated using the abbreviated MDRD (Modification of Diet in Renal Disease). - Mg was analyzed as a continuous, based on the lower and upper normal limits of the laboratory (3 groups: <1.8 mg/dL, 1.8–2.2 mg/dL, and >2.2 mg/dL).	- Low serum Mg levels predict higher mortality in CKD, independent of the initial degree of renal impairment. - Mg concentrations were related to the rate of kidney function decline after adjustment for age, sex, diabetes, and hypertension. - Low serum Mg predicts a faster decline in kidney function.
Sakaguchi et. al. [58]	2013	Observational cohort study	- 66.0 ± 12.5 years. - 88,290 (61.9%) were male.	- 142,555 patients in the nationwide registry of patients with End Stage Renal Disease in Japan.	- Between 2009 and 2010. - 1-year follow-up of all-cause and cause-specific mortality.	- The mean serum Mg level was 2.61 (0.52) mg/dl.	- Lower serum Mg level was a significant and independent predictor of cardiovascular mortality among the chronic hemodialysis population. - Lower Mg level was significantly associated with older age, lower albumin, calcium, phosphate, and hemoglobin level, higher C-reactive protein and alkaline phosphate level, increased prevalence of diabetes mellitus, prior history of cardiovascular disease, and hip fracture.
Lacson et. al. [68]	2015	Observational retrospective cohort study.	- 61.7 ± 14.8 years. - 11, 650 (54.1%) were male. - Follow-up analysis: 61.8 ± 14.8 years and 14,799 (53.7%) were male.	- 21,534 patients of Fresenius Medical Care North America outpatient dialysis facilities. - Follow-up analysis: *n* = 27,544 patients.	- April 2007 through June 2008. - 1-year follow-up.	- Hypomagnesemia: Mg < 1.30 mEq/L; low, mid, and high-normal Mg levels: 1.30 to < 1.60, 1.60 to < 1.90, and 1.90 to 2.10 mEq/L, respectively. - Hypermagnesemia: >2.10 mEq/L. - The mid-normal range (1.60 to <1.90 mEq/L) was used as the reference group.	- The mean serum Mg level was higher overall that the prescribed dialysate mg concentration, with a positive correlation (PC: R = 0.22; *p* < 0.001). - Increasing serum Mg levels were associated with decreasing 1-year mortality risk. - Patients with serum Mg levels > 2.10 mEq/L had a survival advantage (HR, 0.89; 95% CI, 0.80–0.95).
Rebholz et. al. [69]	2016	Prospective cohort study.	- 47 years. - 41% were male.	- 1252 HANDLS study participants (Baltimore, Maryland, USA).	- August 2004–March 2009.	- **Dietary Mg intake** was 116 (96–356) mg/1000 kcal. - The mean baseline eGFR in the overall study population was 97 mL/min/1.73 m^2^. - eGFR was calculated using the CKD- EPI equation.	- Increased probability of rapid deterioration of renal function in association with low dietary intake of Mg (eGFR 100 vs. 94 mL/min /1.73 m^2^; *p* < 0.001). - Dietary intake of Mg was associated with rapid kidney function decline independent of multiple kidney disease risk factors.
Ferrè et. al. [70]	2017	A multiethnic, population-based, cohort study.	- 30–65 years. - 47.2% were male.	- 3551 participants from Dallas County, USA. - Non- CKD (*n* = 3245) - CKD (*n* = 306)	- Between 2007 and 2009. - Subjects followed over a median period of 12.3 years.	- Serum Mg was normally distributed with a mean ± SD value of 2.07 ± 18 mg/dL (0.85 ± 0.07 mM) in the entire cohort, and 2.08 ± 0.19 mg/dL (0.85 ± 0.08 mM) in the CKD and 2.07 ± 0.18 mg/dL (0.85 ± 0.07) in the non-CKD subgroups. - eGFR was calculated using the CKD- EPI equation and the MDRD.	- Low serum Mg levels are independently associated with a higher risk of all-cause death in patients with prevalent early-stage CKD. - And is a significant predictor of death in pre-dialysis CKD patients or patients undergoing hemodialysis.
Farhadnejad et. al. [71]	2016	Prospective population-based cohort study.	- 43.3 ± 11.4 years. - 49.2% were men.	- 1692 patients from 3 medical health centers in Tehran, Iran. - Non- CKD: *n* = 1519; CKD: *n* = 173.	- From 2006 to 2008. - During a median follow-up of 3.6 years.	- CKD was defined as eGFR < 60 mL/min//1.73 m^2^. - eGFR was calculated using the abbreviated MDRD (Modification of Diet in Renal Disease). - **Dietary intakes** were collected using a food frequency questionnaire.	- Higher intakes of Mg (OR: 0.41, 95% CI: 0.22–0.76) were significantly associated with a lower risk of CKD. - The protective effect of Mg (estimated average requirement: 581 mg) decreases the risk of CKD by 60%.
Azem et. al. [63]	2020	Observational cohort study.	- 68.7 ± 13.3 years. - 47% were men.	- 10,568 patients from the Cleveland Clinic CKD registry, USA)	- From 2005 to December 2014. - During a median follow-up of 3.7 years.	- The mean eGFR of the study population was 46.3 mL/min/1.73 m^2^. - eGFR was calculated using the CKD- EPI equation. - Serum Mg was classified based on the normal range into the following categories: <1.7, 1.7–2.6, and >2.6 mg/dL. - Mg data obtained within one year prior to the second e - GFR < 60 mL/min/1.73 m^2^ was included.	- U-shaped association between serum Mg levels and mortality, with both hypomagnesemia and hypermagnesemia (HR = 1.23, 95% CI: 1.03, 1.48). - No association between serum Mg levels and the rate of eGFR decline in CKD patients.
Galán Carrillo et. al. [64]	2021	Retrospective observational cohort study.	- 70 ± 13 years. - 62.9% were male.	- 746 patients with CKD in a nephrology outpatient unit in Spain. - CKD grade 3: 45.2%; CKD grade 4: 35.9% (not on dialysis).	- Between December 2010 and December 2012. - Followed up to December 2016. - Were followed for a mean of 42.6 months.	- The mean serum Mg concentration was 2.09 ± 0.33 mg/dL. - eGFR was calculated using the CKD-EPI equation.	- The use of calcitriol (*p* = 0.029) was associated with higher serum Mg concentration, while calcium supplements (*p* = 0.038) and proton pump (*p* = 0.026) inhibitors were associated with lower serum Mg concentration. - Loop diuretics demonstrated a statistically significant positive association with serum Mg (*p* < 0.001). - No association was found between serum Mg concentration and initiation of kidney replacement therapy. - Patients with hypermagnesemia (Mg > 2.2 mg/dL) had a higher risk of cardiovascular events (*p* = 0.028).

## 4. Discussion

The role of Mg in renal function is supported by all the articles included and analyzed in this review. Furthermore, this relationship is also observed in patients with kidney disease as well as in healthy elderly patients [72,73].

For patients without kidney disease, maintaining a healthy intake of a micronutrient-rich diet reduces the risk of renal failure [71]. For example, it has been proven that hypomagnesemia can lead to an inflammatory, atherogenic, and thrombotic response in the vasculature [74]. Also, it may affect the healing process after vascular injury by regulating endothelial cell migration and proliferation [75]. Additionally, higher Mg intake through oral supplementation can lower blood pressure levels, which is an established risk factor for kidney disease [69,76].

On the other hand, the progression of CKD leads to alterations in mineral metabolism, and as such, its severity increases as the disease advances. Older age has been associated with the occurrence and higher progression of CKD [77]. Moreover, it has also been proven that among CKD patients, hypomagnesemia can increase the risk of progression to end-stage renal disease (ESRD) and cardiovascular disease [67,68,78].

This might be explained by the protective role the Mg exerts in the prevention of arrhythmia and atherosclerosis in ESRD patients [79,80]. Therefore, as Kanbay et. al. proposed, Mg can be used as an important adjuvant for the management of endothelial dysfunction and cardiovascular disease associated with CKD [57].

Also, there are several studies [81,82,83] that show that Mg could provide better clinical outcomes by preventing the progression of vascular calcification in CKD.

Another important association proposed in this review is the one of Mg concentration with risk of mortality in CKD patients [58,66]. We found that higher Mg levels are beneficial in terms of survival. In support of that, Van Laecke et al. confirm an association between hypomagnesemia with an increased risk for death and accelerated kidney function deterioration [67].

Furthermore, a significant association has been shown between a higher Mg intake and a lower incidence of type 2 diabetes mellitus [84] due to the role that Mg has in the improvement of insulin resistance and beta-cell function [85,86].

Nevertheless, there are still two important questions that need to be addressed to establish an optimal clinical practice: What is the correct target range of serum Mg levels and could it be standardized for every patient? What is the best approach to increase serum Mg levels? The answers to these questions could help to define an optimal strategy to manage Mg-related complications and improve the prognosis of CKD patients [87].

Quality of life is another important aspect to take into consideration. Mobility and pain discomfort are the two aspects in patients with moderate renal dysfunction (eGFR of 30.0–59.9 mL/min/1.73 m^2^) [88]. This not only affects the quality of daily life by making them more dependent patients but also increases the risk of mortality and leads to more comorbidities.

Regarding sex differences, it has been shown that the parenchymal kidney volume in males increased up to middle age and then progressively declined, more sharply after 70 years of age, whereas females trended towards a gradual decrease in kidney volume through life [44,47]. This can be explained by the kidney sex hormone receptors. In a study conducted in an elderly cohort [89], it was shown that Mg levels are strongly and independently associated with the anabolic hormones, testosterone and IGD-1. Therefore, in terms of eGFR, chronic testosterone deficiency may lead to reduced kidney function [90].

Finally, there was only one article [67] in this review that found an increased incidence of hypermagnesemia in men. Making an association with higher dietary intake of Mg in contrast with women, which can be related to socioeconomic factors [91].

## 5. Conclusions

Magnesium is a relevant cation that needs further research to expand the knowledge of its implications and have a better understanding of its preventing role.

Altogether, we conclude that well-designed prospective clinical trials are needed to determine in which cases there would be an indication to recommend Mg supplementation, either dietary or pharmacological, taking age and sex into account.
